# Transactions of the New Hampshire Medical Society

**Published:** 1856-01

**Authors:** 


*Transactions of the New Hampshire Medical Society*, for 1855.

This contains a most interesting address, by J. S. Fernald, M. D., and President of the Society. This is followed by a dissertation on "Progressive Medicine," by A. H. Robinson, M. D., which is ably written; another on Fracture, by W. P. Stone, M. D.; then a report on Hernia, which is followed by another upon the examination at Dartmouth College.

